# The Indispensable Role of Cyclin-Dependent Kinase 1 in Skeletal Development

**DOI:** 10.1038/srep20622

**Published:** 2016-02-10

**Authors:** Masanori Saito, Mieradili Mulati, S. Zakiah A. Talib, Philipp Kaldis, Shu Takeda, Atsushi Okawa, Hiroyuki Inose

**Affiliations:** 1 Department of Orthopedics, Graduate School, Tokyo Medical and Dental University, 1-5-45 Yushima, Bunkyo-ku, Tokyo 113-8519, Japan; 2 Institute of Molecular and Cell Biology (IMCB), A*STAR (Agency for Science, Technology and Research), 61 Biopolis Drive, Proteos#3-09, Singapore 138673, Republic of Singapore; 3 National University of Singapore (NUS), Department of Biochemistry, Singapore 117597, Republic of Singapore; 4 Department of Physiology and Cell Biology, Tokyo Medical and Dental University, 1-5-45 Yushima, Bunkyo-ku, Tokyo 113-8519, Japan

## Abstract

Skeletal development is tightly regulated through the processes of chondrocyte proliferation and differentiation. Although the involvement of transcription and growth factors on the regulation of skeletal development has been extensively studied, the role of cell cycle regulatory proteins in this process remains elusive. To date, through cell-specific loss-of-function experiments *in vivo*, no cell cycle regulatory proteins have yet been conclusively shown to regulate skeletal development. Here, we demonstrate that cyclin-dependent kinase 1 (Cdk1) regulates skeletal development based on chondrocyte-specific loss-of-function experiments performed in a mouse model. Cdk1 is highly expressed in columnar proliferative chondrocytes and is greatly downregulated upon differentiation into hypertrophic chondrocytes. Cdk1 is essential for proper chondrocyte proliferation and deletion of Cdk1 resulted in accelerated differentiation of chondrocytes. *In vitro* and *ex vivo* analyses revealed that Cdk1 is an essential cell cycle regulatory protein for parathyroid hormone-related peptide (PTHrP) signaling pathway, which is critical to chondrocyte proliferation and differentiation. These results demonstrate that Cdk1 functions as a molecular switch from proliferation to hypertrophic differentiation of chondrocytes and thus is indispensable for skeletal development. Given the availability of inhibitors of Cdk1 activity, our results could provide insight for the treatment of diseases involving abnormal chondrocyte proliferation, such as osteoarthritis.

Skeletal development begins with the formation of mesenchymal condensations. Mesenchymal cells differentiate into round chondrocytes to form the cartilage template. The primary ossification center then develops and expands to convert the central segment into bone tissue, leaving a region of cartilage at each end. The resulting cartilage is organized such that the distal portion of the cartilage contains round, proliferating chondrocytes. Towards the center, the round chondrocytes differentiate into flat columnar proliferative chondrocytes, and those that are close to the primary ossification center stop proliferating and differentiate into post-mitotic hypertrophic chondrocytes. Finally, the hypertrophic chondrocytes start secreting a matrix rich in collagen type X, direct the mineralization of the surrounding matrix, and attract blood vessels and chondroclasts to remodel cartilage into bone^1^,^2^. Thus, during the course of skeletal development, the architecture of the growth plate is strictly regulated through a balance between chondrocyte proliferation and differentiation^2^,^3^. Any abnormality in this regulation results in a disorganized growth plate, which leads to pathological skeletal conditions such as osteochondrodysplasias.

To date, the involvement of growth and transcription factors in skeletal development has been extensively studied^2^,^3^,^4^. Among those factors, parathyroid hormone-related peptide (PTHrP) and Indian hedgehog (Ihh) have been demonstrated to be central regulators of chondrocyte proliferation and differentiation^5^,^6^. In skeletal development, PTHrP expression is high in the periarticular resting chondrocytes and is low in the proliferating chondrocytes^7^. PTHrP works as a paracrine factor in the growth plate, maintaining chondrocytes in a proliferative state and delaying terminal chondrocyte differentiation^2^. The columnar proliferative chondrocytes that are located at a sufficient distance from the PTHrP source withdraw from the cell cycle and initiate terminal differentiation into hypertrophic, Ihh-synthesizing cells. Ihh is synthesized by pre-hypertrophic chondrocytes, stimulates the production of PTHrP in resting chondrocytes^2^, and regulates chondrocyte differentiation and proliferation through both PTHrP-dependent and -independent pathways^8^. Therefore, PTHrP and Ihh form a negative feedback loop that controls the site of post-mitotic–hypertophic differentiation and the length of the columnar proliferating chondrocytes^2^. Since PTHrP and Ihh both regulate chondrocyte proliferation^2^,^3^, they must directly or indirectly regulate the cell cycle machinery. However, the precise mechanism by which these factors regulate the cell cycle machinery and the specific cell cycle regulators involved remain unknown.

The cell cycle is regulated by cell cycle regulatory proteins such as cyclins, cyclin-dependent kinases (Cdks), and cyclin-dependent kinase inhibitors (CKIs)^9^. Cyclins possess no enzymatic activity, but activate Cdks by direct binding. These Cdk/cyclin complexes then activate downstream cell cycle proteins that are essential for initiating the next cell cycle phase. By contrast, CKIs negatively regulate Cdks by direct binding to Cdk/cyclin complexes^9^. Among the many cell cycle proteins, we have chosen to specifically focus on the involvement of Cdks during skeletal development, since they function as the primary engine of the cell cycle^9^.

The mammalian genome contains at least 20 different Cdk-encoding genes, and widespread compensatory mechanisms among them have been reported^10^. Indeed, conventional knockout mice of Cdk2, Cdk4, or Cdk6 were reported to be viable, and do not show any overt skeletal phenotypes^11^,^12^. Therefore, these Cdk genes are unlikely to play a major role in skeletal development, at least during embryonic development. Cdk1 was the first Cdk gene identified, and is conserved in all organisms^10^. However, the physiological role of Cdk1 in skeletal development remains unknown, due to the fact that its deletion leads to embryonic lethality^10^. Despite its theoretical importance, no cell cycle regulatory proteins have been identified to regulate skeletal development through cell-specific loss-of-function experiments conducted *in vivo*. Here, we show that Cdk1 is highly expressed in columnar proliferating chondrocytes and is downregulated upon differentiation into hypertrophic chondrocytes. Deletion of Cdk1 in chondrocytes physiologically inhibited proliferation and resulted in accelerated differentiation both *in vivo* and *in vitro*. Furthermore, Cdk1 was demonstrated to be an essential downstream factor for the PTHrP signaling pathway. Thus, to our knowledge, this study is the first to demonstrate through Cdk1 that critical regulation of skeletal development by cell cycle regulatory protein through chondrocyte-specific loss-of-function experiments *in vivo*.

## Results

### Cdk1 is indispensable for skeletal development

**Figure 1:**
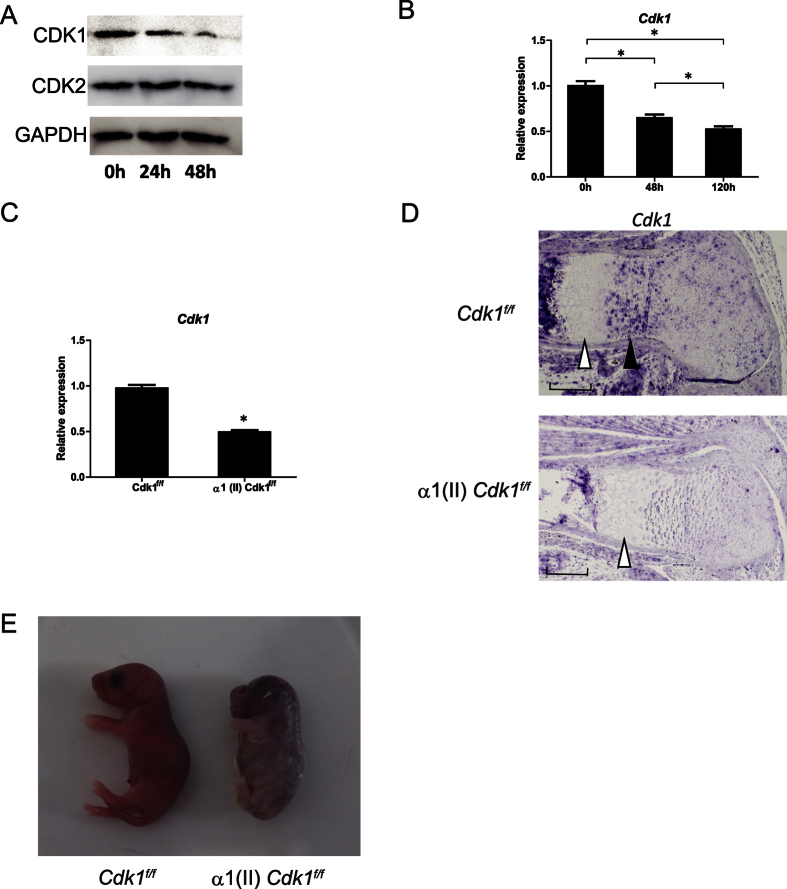
Expression of *Cdk1* during chondrocyte differentiation. (**a**,**b**) Change in the expression of Cdks during chondrocyte differentiation as determined at the protein level with Western blot analysis (**a**) and the mRNA level with qPCR analysis (**b**). ATDC5 cells were cultured in differentiation medium for the indicated lengths of time. Note the significant decrease of *Cdk1* in parallel with the progression of chondrocyte differentiation. *P < 0.05 vs. indicated time point, n = 4. (**c**) *Cdk1* expression in α1(II) *Cdk1*^*f*/*f*^ mice based on qPCR analysis. Growth plate chondrocytes were isolated from *Cdk1*^*f*/*f*^ (control) or α1(II) *Cdk1*^*f*/*f*^ mice and used for subsequent analyses. Note the significant decrease of *Cdk1* expression in α1(II) *Cdk1*^*f*/*f*^ mice. *P < 0.05, n = 3. (**d**) *In situ* hybridization analysis of growth plate sections of E16.5 *Cdk1*^*f*/*f*^ (control) and α1(II) *Cdk1*^*f*/*f*^ mice femur. The section was hybridized with *Cdk1* probes. Note the high expression of *Cdk1* in columnar proliferating chondrocytes (black arrowhead) and the low expression in hypertrophic chondrocytes (white arrowhead). Scale bar: 100 μm. (**e**) Gross morphology on P0. Representative α1(II) *Cdk1*^*f*/*f*^ neonates are shown with shorter limbs and body as compared to their *Cdk1*^*f*/*f*^ littermates. All α1(II) *Cdk1*^*f*/*f*^ mice died soon after birth.

To investigate the Cdks that are potentially involved in chondrocyte development, we first examined the levels of Cdk proteins during chondrocyte differentiation *in vitro*. The levels of the Cdk proteins were confirmed in ATDC5 cells, a murine chondrocyte cell line, by Western blot analysis. The protein levels of Cdk2 were unchanged during chondrocyte differentiation, confirming previous reports^13^, whereas the protein levels of Cdk1 gradually decreased over the course of differentiation (Fig. 1a). To quantify *Cdk1* expression at the transcriptional level, we performed a quantitative real-time polymerase chain reaction (qPCR) analysis and found that the mRNA level was also decreased during chondrocyte differentiation (Fig. 1b). Since *Cdk1*-null mice tend to die around embryonic day 3.5 (E 3.5)^10^, we generated chondrocyte-specific knockout mice to be able to investigate the role of *Cdk1* during skeletal development. To achieve this, we crossed *Cdk1f*/*f* (hereafter, “control”) mice with transgenic mice expressing Cre recombinase under the control of the α1 (II)-collagen promoter (hereafter, “α1 (II) Cre mice”) to generate α1 (II)-Cre tg/*Cdk1f*/*f* mice (hereafter, “α1 (II) *Cdk1*^*f*/*f*^ mice”)^10^,^14^. These mutant mice were recovered in the expected Mendelian ratio. The deletion of *Cdk1* was confirmed in the growth plate chondrocytes by qPCR (Fig. 1c) and *in situ* hybridization (Fig. 1d). *In situ* hybridization of the control mouse femur sections revealed moderate expression of *Cdk1* in the round proliferative chondrocytes and high expression in the columnar proliferative chondrocytes, whereas the expression was greatly diminished in post-mitotic hypertrophic chondrocytes within the growth plate (Fig. 1d). Interestingly, α1 (II) *Cdk1*^*f*/*f*^ mice were significantly smaller than their control littermates at P0 (Fig. 1e and S1). However, α1 (II) *Cdk1*^*f*/*f*^ mice died shortly after birth, probably due to respiratory problems.

### Cdk1 is indispensable for the maintenance of chondrocyte proliferation and differentiation in the growth plate

**Figure 2:**
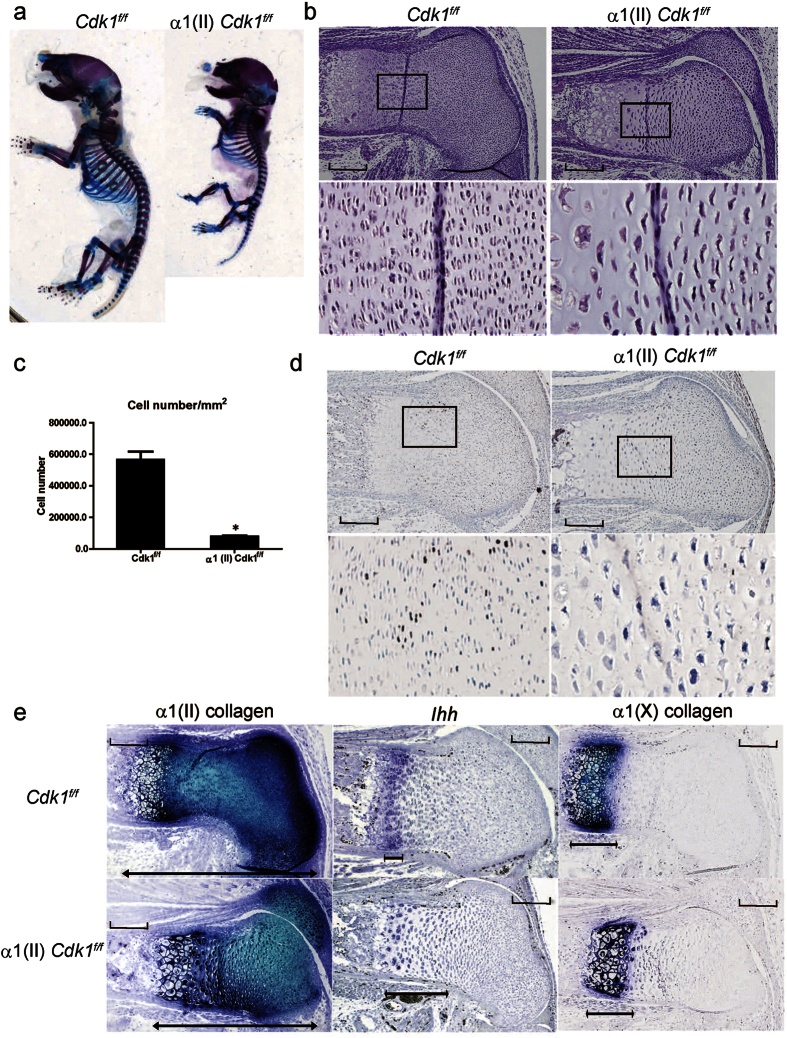
Skeletal phenotypes of Cdk1 conditional knockout mice. (**a**) Skeletal staining with Alcian Blue and Alizarin Red on P0. Note the significantly shorter limbs in the α1(II) *Cdk1*^*f*/*f*^ neonates and lack of pattering defects. (**b**) Hematoxylin and eosin-stained sections of the femurs obtained on E16.5. Severe defects were found in the growth plate cartilage of α1(II) *Cdk1*^*f*/*f*^ mice, characterized by the loss of columnar proliferating chondrocytes and early transition into pre-hypertrophic chondrocytes. Boxed regions are magnified beneath to show the columnar proliferative chondrocytes. Scale bar: 100 μm. (**c**) Chondrocytes were counted at columnar proliferative layers. The number of proliferative chondrocytes in α1(II) *Cdk1*^*f*/*f*^ mice is significantly reduced. *P < 0.01, n = 3. (**d**) Chondrocyte proliferation in wild-type and α1(II) *Cdk1*^*f*/*f*^ mice at E16.5 was examined by BrdU labeling. Boxed regions are magnified beneath to show BrdU-positive cells. Note that the BrdU-positive cells were largely eliminated in α1(II) *Cdk1*^*f*/*f*^ mice. Scale bar: 100 μm. (**e**) *In situ* hybridization analysis of sections from the femur growth plate obtained on E16.5. Adjacent sections were hybridized with α1(II) collagen (left), *Ihh* (middle), and α1(X) collagen (right) probes. Note the contraction of the growth plate as indicated by the shortening of the α1(II) collagen domain, whereas there is no contraction of the α1(X) collagen domain that marks hypertrophic chondrocytes and expansion of the *Ihh* domain that marks pre-hypertrophic chondrocytes in the growth plate of α1(II) *Cdk1*^*f*/*f*^ mice. Scale bar: 100 μm.

Skeletal preparations of the pups of α1(II) *Cdk1*^*f*/*f*^ mice revealed that deletion of Cdk1 resulted in significantly shortened limbs and rounded skulls (Fig. 2a). Histological analysis of the femur revealed the absence of columnar proliferative chondrocytes (Fig. 2b). In addition, the diameter of Cdk1KO chondrocytes increased by 50%, and the cell density was decreased by 91% compared to their control counterparts, which is consistent with the ablated chondrocyte cell division and substantial increase in cell growth observed in Cdk1KO chondrocytes. Furthermore, the mice with Cdk1 deletion had significantly lower numbers of pre-hypertrophic and hypertrophic chondrocytes compared to their control littermates (Fig. 2c). BrdU labeling was performed to investigate whether chondrocyte proliferation was inhibited in the mutant mice. Indeed, the proliferative chondrocytes, i.e., BrdU-positive cells, were largely eliminated in the possible columnar proliferating zone in α1(II) *Cdk1*^*f*/*f*^ mice (Fig. 2d). These results confirm that Cdk1 is indispensable for chondrocyte proliferation *in vivo*.

The abnormal growth plate of the α1 (II*) Cdk1*^*f*/*f*^ mice further prompted us to perform *in situ* analysis for chondrocyte differentiation markers to investigate the physiological role of Cdk1 in chondrocyte differentiation. The length of the growth plate, characterized by the expression of α1(II) collagen, was decreased in the α1(II) *Cdk1*^*f*/*f*^ mice, whereas the length of the hypertrophic zone, characterized by the expression of α1 (X) collagen, showed no difference between the mutant and control mice (Fig. 2e). Importantly, the pre-hypertrophic zone, characterized by the expression of *Ihh*, was wider and located closer to the epiphysis in α1(II) *Cdk1*^*f*/*f*^ mice (Fig. 2e). Since chondrocytes begin to exit the cell cycle during the transition from proliferative to pre-hypertrophic chondrocytes^15^, these results indicated that deletion of Cdk1 causes the chondrocytes to exit the cell cycle, resulting in accelerated differentiation and maturity into pre-hypertrophic chondrocytes. However, Cdk1 does not appear to be required for terminal differentiation in developing bone, as the last steps in this process, such as replacement of hypertrophic chondrocytes with cancellous bone, occurred in the absence of Cdk1. Interestingly, the number of apoptotic hypertrophic chondrocytes was comparable between α1(II) *Cdk1*^*f*/*f*^ mice and their wild-type littermates (Fig. S2), suggesting that Cdk1 has little effect on chondrocyte apoptosis in the growth plate. These findings suggest that loss of Cdk1 results in a reduction of proliferating chondrocytes via decelerated proliferation and accelerated differentiation.

### Knockdown of Cdk1, not Cdk2, resulted in reduced chondrocyte proliferation and accelerated chondrocyte differentiation *in vitro*

**Figure 3:**
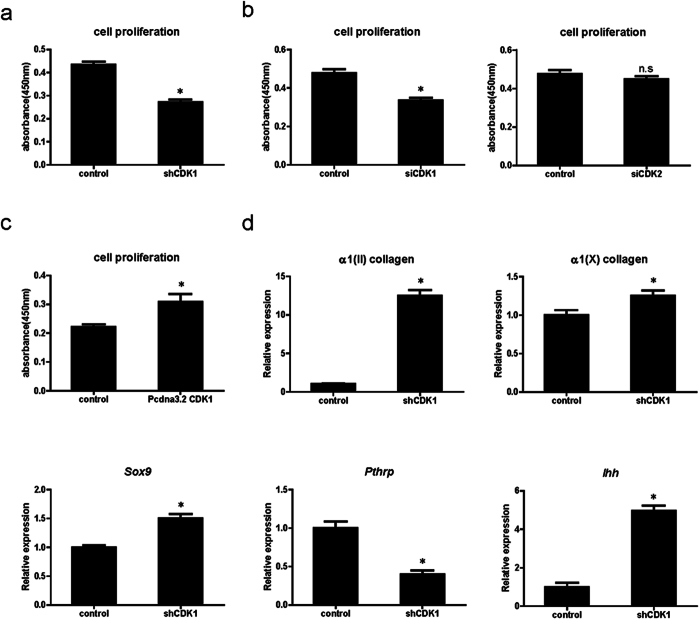
Knockdown of *Cdk1* in chondrocytes inhibits proliferation and promotes differentiation *in vitro.* (**a**,**b**) Effect of *Cdk1* knockdown on chondrocyte proliferation: ATDC5 cells constitutively expressing shRNA against *Cdk1* (**a**) or transiently transfected with siRNA against *Cdk1* or *Cdk2* (b). Note the significant decrease in proliferation induced by the *Cdk1* knockdown. n.s.: not significant. *P < 0.05, n = 4. (**c**) Effect of *Cdk1* overexpression on chondrocyte proliferation: ATDC5 cells were transfected with *Cdk1* or control. Note the significant increase in proliferation of *Cdk1*-expressing cells. *P < 0.05, n = 4. (**d**) Effect of continuous inhibition of *Cdk1* on chondrocyte differentiation: ATDC5 cells infected with sh Cdk1 or a control retrovirus were cultured and subjected to qPCR analysis for the indicated genes. Note the significant elevated expression of chondrocyte differentiation markers by the stable knockdown of *Cdk1. Gapdh* was used as an internal control. *P < 0.05, n = 4.

The observed inhibition of chondrocyte proliferation in α1(II) *Cdk1*^*f*/*f*^ mice prompted us to test whether inhibition of Cdk1 would also affect chondrocyte proliferation *in vitro*. To this end, we infected ATDC5 cells with a vector expressing small hairpin RNA (shRNA) against *Cdk1* and isolated stable puromycin-resistant clones. As a control, we also infected cells with shRNA against the luciferase gene. The knockdown efficiency was confirmed by qPCR (Fig. S3). Indeed, the proliferation of ATDC5 cells with stably knocked down *Cdk1* expression was significantly impaired compared to control shRNA-transfected cells (Fig. 3a). To rule out the possibility that stable expression of shRNA against Cdk1 itself altered the properties of ATDC5 cells, we also transiently transfected small interfering RNA (siRNA) against *Cdk1* or *Cdk2* into ATDC5 cells. In this transient siRNA transfection assay, knockdown of Cdk2 did not affect chondrocyte proliferation, whereas knockdown of Cdk1 did (Fig. 3b). By contrast, overexpression of Cdk1 significantly promoted the proliferation of ATDC5 cells (Fig. 3c). Taken together, these results demonstrate that Cdk1, but not Cdk2, physiologically regulates chondrocyte proliferation. Next, we examined the effect of Cdk1 knockdown on chondrocyte differentiation *in vitro*. In line with our observations *in vivo*, knockdown of Cdk1 resulted in accelerated chondrocyte differentiation as confirmed by the elevated expression of chondrocyte differentiation markers [α1(II) collagen, α1(X) collagen, and *Sox9*] (Fig. 3d). Collectively, these results also confirmed that the deletion of Cdk1 results in inhibition of chondrocyte proliferation and acceleration of chondrocyte differentiation *in vitro*. Taken together, these results indicate that Cdk1 is not only essential for the proliferation but also for the differentiation of chondrocytes, by regulating their exit from the cell cycle.

### Cdk1 is an essential cell cycle regulatory protein involved in PTHrP signaling

**Figure 4:**
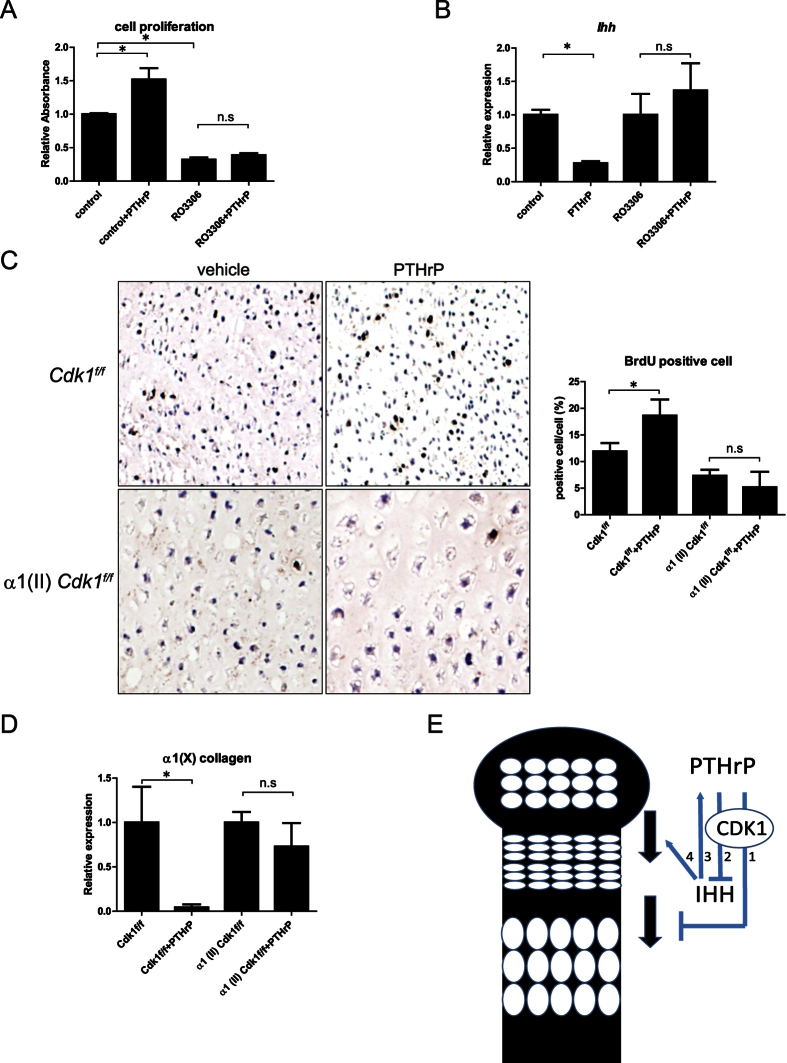
Cdk1 is an essential cell cycle regulator downstream of PTHrP signaling. (**a**) Effect of PTHrP on chondrocyte proliferation in ATDC5 cells treated with RO-3306: ATDC5 cells were treated with combination of RO-3306 followed by PTHrP. Then a cell proliferation assay was performed. PTHrP treatment increased proliferation in controls, whereas this effect was abolished by inhibition of Cdk1 activity. n.s.: not significant. *P < 0.05, n = 4. (**b**) qPCR analysis showing *Ihh* mRNA levels in ATDC5 cells treated with RO-3306 and PTHrP. PTHrP treatment reduced *Ihh* expression in controls, whereas this effect was abolished by inhibition of Cdk1 activity. n.s.: not significant. *P < 0.05, n = 4. (**c**) Chondrocyte proliferation of PTHrP-treated and vehicle-treated control and α1(II) *Cdk1*^*f*/*f*^ limb explants examined by BrdU labeling. The number of BrdU-positive and -negative cells in the proliferating chondrocytes were counted. Note that deletion of *Cdk1* blunted the effect of PTHrP on proliferation. n.s.: not significant. *P < 0.05, n = 3. (**d**) Chondrocyte differentiation of PTHrP-treated and vehicle-treated control and α1(II) *Cdk1*^*f*/*f*^ limb explants examined by qPCR analysis. Note that deletion of *Cdk1* blunted the repressive effect of PTHrP on differentiation. n.s.: not significant. *P < 0.05, significant difference from vehicle. n = 3. (**e**) Proposed model of action of Cdk1 in the growth plate. Cdk1 mediates the activity of PTHrP to inhibit chondrocyte terminal differentiation (1), maintain chondrocytes in a proliferative state (1), and delay Ihh production (2). In turn, Ihh positively regulates PTHrP expression (3) and stimulates early chondrocyte differentiation (4).

The reduced proliferation and early transition into pre-hypertrophic chondrocytes in α1 (II) *Cdk1*^*f*/*f*^ mice are similar to the effects previously observed under conditions of PTHrP deficiency^4^,^16^. Given that the PTHrP signaling pathway is an essential regulator of chondrocyte proliferation and differentiation, we examined whether Cdk1 also plays a role in this pathway. To investigate whether Cdk1 is involved in the PTHrP signaling pathway, we treated ATDC5 cells with RO-3306, a small-molecule specific inhibitor of Cdk1 activity^17^, followed by recombinant PTHrP. The recombinant PTHrP failed to rescue the reduced proliferation caused by the pharmacological inhibition of Cdk1 activity in ATDC5 cells (Fig. 4a). In addition, the treatment with recombinant PTHrP also failed to downregulate *Ihh* expression in Cdk1 activity-inhibited ATDC5 cells (Fig. 4b), suggesting the potential involvement of Cdk1 activity in the downstream PTHrP signaling pathway. Next, to investigate whether Cdk1 mediates the effects of PTHrP on chondrocyte proliferation and differentiation, we designed an experiment based on an organ culture system, since α1(II) *Cdk1*^*f*/*f*^ mice were not viable and the basal expression level of PTHrP in the cartilage was too low^18^ to reliably detect its possible downregulation in α1(II) *Cdk1*^*f*/*f*^ mice. The organ culture system is an established *ex vivo* model for chondrocyte proliferation and differentiation, which retains normal patterns of chondrocyte proliferation and differentiation for at least 4 days in culture^4^. Treatment with recombinant PTHrP induced chondrocyte proliferation, as shown by the markedly increased number of BrdU-positive cells in the growth plate of control hind limbs; however, the effect of PTHrP was blunted in α1(II) *Cdk1*^*f*/*f*^ hind limbs (Fig. 4c). These results suggest that activation of the PTHrP signaling pathway in α1(II) *Cdk1*^*f*/*f*^ limbs cannot rescue the defects in chondrocyte proliferation, which is characteristic of this mutant. Next, to determine how PTHrP regulates growth plate chondrocyte differentiation in Cdk1-deficient limbs, we compared the effect of PTHrP on chondrocyte differentiation using qPCR. As expected, PTHrP treatment resulted in a significant decline in the expression of α1(X) collagen, a hypertrophic chondrocyte marker, in control mice. However, the repressive effect was blunted in the α1(II) *Cdk1*^*f*/*f*^ hind limbs, suggesting that PTHrP’s effect on chondrocyte differentiation is, at least in part, Cdk1 dependent (Fig. 4d). Taken together, these results suggest that Cdk1 plays an important role in how PTHrP regulates chondrocyte proliferation and differentiation in the growth plate.

## Discussion

Cdk1 is a 34-kDa protein that functions as a serine/threonine kinase, and forms complexes with its partners cyclin A and cyclin B to phosphorylate target substrates and progress through the cell cycle^9^. We showed that Cdk1 is highly expressed in columnar proliferating chondrocytes and is greatly downregulated upon differentiation into hypertrophic chondrocytes. Cdk1 is required for proper chondrocyte proliferation and deletion of Cdk1 resulted in accelerated differentiation of chondrocytes *in vivo* and *in vitro*. Cdk1 acts as an essential downstream factor for PTHrP signaling in chondrocyte proliferation and differentiation. These results suggest that Cdk1 is an important cell cycle regulatory protein in the PTHrP-dependent regulation of chondrocyte development, and thus plays a critical role in skeletal development (Fig. 4e).

With respect to the role of Cdks in the regulation of skeletal development, recent studies demonstrated the involvement of Cdk6 in chondrocyte differentiation^19^ and apoptosis^13^. However, these reports were based on *in vitro* and gain-of-function experiments. In addition, transgenic mice overexpressing Cdk6 in chondrocytes showed no skeletal abnormality^19^, and Cdk6 knockout mice did not display gross anatomical abnormality^12^. Other cell cycle factors such as cyclin D1 and p21 have also been shown to play a role in chondrocyte proliferation^20^. Cyclin D1 acts downstream of PTHrP and transforming growth factor-β to promote chondrocyte proliferation^21^; cyclin D1-deficient mice show reduced postnatal growth^21^, and p21 was found to be involved in the effect of fibroblast growth factor on growth inhibition of the cartilage^22^. However, excluding mice lacking p57, single-knockout mice of cyclin D1,p21, and other major Cdks and CKIs appeared to be indistinguishable from their wild-type littermates at birth^11^,^12^,^21^,^23^,^24^,^25^, suggesting the existence of compensatory mechanisms over these genes. By contrast, the results of the present study revealed that chondrocyte-specific deletion of Cdk1 caused severe dwarfism at birth. These results suggest that, at least during embryonic development, the previously mentioned cell cycle proteins have less of an effect on the regulation of chondrocyte development than does Cdk1. Thus, our study revealed Cdk1 as a key cell cycle regulatory protein in skeletal development.

Mice lacking the CKI p57 also display the same short limbs comparable to those observed in the α1(II) *Cdk1*^*f*/*f*^ mice^26^. This abnormality is due to the delayed differentiation and increased proliferation of chondrocytes^26^, which is the reverse image of the effects occurring in α1(II) *Cdk1*^*f*/*f*^ mice. In addition, the expression of p57 is low in proliferating chondrocytes, whereas hypertrophic chondrocytes express high levels of p57. Considering that p57 is a potent inhibitor of all G1/S-phase Cdks and Cdk1^27^, then expression of p57 should inhibit Cdk1 to trigger differentiation in stem cells^28^. Thus the findings that high Cdk1 expression in the columnar proliferative chondrocytes and low Cdk1 expression in the hypertrophic chondrocyte suggest that an increase in p57 would also inhibit Cdk1 expression to trigger hypertrophic differentiation in differentiating chondrocytes. However, the specific interaction between Cdk1 and p57 in chondrocytes remains to be elucidated.

In this study, the expression of *Ihh* was preserved in the growth plates of mutant mice and Cdk1-knockdown cells (Figs 2e and 3d), suggesting that the reduction of chondrocyte proliferation and the acceleration of chondrocyte differentiation observed in α1(II) *Cdk1*^*f*/*f*^ mice were caused by defects downstream or independent of the Ihh signaling pathway. The PTHrP signaling pathway is another critical downstream effector of Ihh that regulates chondrocyte proliferation and differentiation^2^. Considering our present observations, we hypothesize that Cdk1 regulates the factors downstream of PTHrP signaling such as PTHR1 or PTHrP itself. Since PTHR1 is phosphorylated to work as an effector for acute PTHrP signaling^29^, it is possible that Cdk1 also regulates the activity of PTHR1 or other essential downstream molecules for proper PTHrP signaling. Indeed, there are two putative phosphorylation motifs (70T and 519S) for Cdk1 in the PTHR1 amino acid sequence. In addition, PTHrP itself has been found to be phosphorylated by Cdk1 at residue 85T immediately preceding the nuclear localization sequence (NLS), localizes to the nucleus at the G1 phase of cell cycle, and to be transported to the cytoplasm when cells divide^30^. Since the intracellular localization of PTHrP is both phosphorylation- and cell cycle-dependent^30^ and deletion of the NLS in PTHrP results in reduced chondrocyte proliferation *in vivo*^31^, loss of Cdk1 in chondrocytes may result in disruption of intracellular PTHrP signaling, which would consequently stop their proliferation, leading to differentiation.

Another interesting finding emerging from this study is that Cdk1 expression was high in proliferating chondrocytes but low in hypertrophic chondrocytes (Fig. 1d). The high level of Cdk1 expression in columnar proliferating chondrocytes suggests that Cdk1 functions to maintain chondrocytes in a proliferating state and delay further differentiation. Accordingly, downregulation of Cdk1 expression in chondrocytes may be important for terminal chondrocyte differentiation, which is in agreement with our observations. However, the specific molecular mechanism contributing to the downregulation of Cdk1 during skeletal development remains to be elucidated. Since Cdk1 is transcriptionally activated in various tissues during embryogenesis^32^, it is hypothesized that transcription factors involved in chondrocyte differentiation regulate Cdk1 expression. Therefore, it is possible that certain transcription factors that are essential for chondrocyte differentiation, such as Sox9 and Runx2, also regulate Cdk1 expression. Indeed, there are many putative binding sites for these factors in the sequence upstream of the Cdk1 locus.

In conclusion, we demonstrated that Cdk1 is required for proper chondrocyte proliferation and differentiation, is essential for key downstream responses to PTHrP, and is thus an important cell cycle protein for skeletal development. Cdk inhibitors are currently being tested for the treatment of various diseases such as breast cancer^33^, chronic lymphocytic leukemia^34^, and rheumatoid arthritis^35^ with promising results. Thus, Cdk1 could also be a potential therapeutic target for the treatment of bone and joint diseases such as osteoarthritis and osteochondrodysplasias by modulating chondrocyte proliferation and differentiation.

## Materials and Methods

### Animals

Cdk1^*f*/*f*^ mice were described previously^10^. α1 (II) Cre mice were described previously^14^. α1 (II) Cre mice were mated with *Cdk1*^*f*/*f*^ mice to obtain chondrocyte-specific Cdk1 knockout mice. We maintained all the mice under standard conditions with food and water available *ad libitum* and maintained on a 12 h light/dark cycle. All animal experiments were performed with the approval of the Animal Study Committee of Tokyo Medical and Dental University and conformed to relevant guidelines and laws.

### *In situ* hybridization

*In situ* hybridization was performed using DIG labeled riboprobe according to the standard protocol as previously described^36^. Hybridizations were performed at 55 °C. For detection, signals were developed using anti –DIG antibody conjugated with alkaline phosphatase. After antibody treatment, sections were incubated with BM Purple (Roche).

### Cell culture

Murine chondrocyte cell line ATDC5 cells were purchased from the Riken Cell Bank (Tsukuba, Japan). The cells were maintained in Dulbecco’s minimal essential medium nutrient mixture F-12 HAM (DMEM/F12; Sigma) containing 5% fetal bovine serum (FBS; Sigma) in 5% CO_2_ in air. For chondrocyte differentiation, ATDC5 cells were treated with chondrogenic medium (5% FBS in the presence of ITS) with or without 10μM RO-3306 (Sigma) to inhibit Cdk1 activity, or 3 × 10^−7^ M recombinant PTHrP (Peptide Institute, Inc) to activate PTHrP signaling. Results are representative of more than four individual experiments.

### BrdU labeling

Pregnant female mice were injected intraperitoneally with 100 μg BrdU/gram body weight and sacrificed 4 hours later. Limbs were dissected and fixed in 4% paraformaldehyde overnight at 4 °C. Tissues were processed, embedded and sectioned using standard procedures. BrdU was detected using a BrdU Staining Kit (Invitrogen).

### Transfection and infection

cDNA fragments of Cdk1 were amplified by PCR. The PCR fragments were cloned into the pcDNA3.2 V5/DEST vector (Invitrogen). For the Cdk1 overexpression study, ATDC cells were seeded and transfected using Lipofectamine LTX reagent (Invitrogen) according to the manufacturer’s instructions. For the Cdk1 knockdown study, ATDC5 cells were seeded and transfected with 20 nM of short interfering Cdk1 (SMARTpool siGENOME Cdk1, Dharmacon), short interfering Cdk2 (SMARTpool siGENOME Cdk2, Dharmacon) or siControl (siGENOME Non-Targeting siRNA pool, Dharmacon) using HiperFect (Qiagen) reagent according to the manufacturer’s instructions. After transfection, cells were cultured with chondrogenic differentiation media.

For the establishment of stable cell lines, we constructed retrovirus-expressing shRNA against Cdk1 using the RNAi-ready pSIREN Vector (Clontech) and Platinum Retroviral Expression System (Cell Biolabs, Inc), as described by the manufacturer. shRNA sequences were 5′-GATCCGTGCCAGAGCGTTTGGAATATTCAAGAGATATTCCAAACGCTCTGGCATTTTTTCTCGAGG-3′and 5′-AATTCCTCGAGAAAAAATGCCAGAGCGTTTGGAATATCTCTTGAATATTCCAAACGCTCTGGCACG-3′ (for *Cdk1*). Stable clones expressing shRNA against Cdk1 or luciferase gene were selected by 5 ug/ml puromycin.

### Cell proliferation assay

The proliferation assay for chondrocytes was performed using the Cell Counting kit-8 (DOJINDO), according to the manufacturer’s instructions. Results are representative of more than three individual experiments.

### Quantitative real-time PCR analysis

Total RNA from tissues and cultured cells was extracted using TRIzol reagent (Invitrogen). Reverse transcription was performed by High capacity cDNA reverse transcription kit (Applied Biosystems) according to the manufacturer’s instructions. We performed quantitative analysis of gene expression using the MX3000p real –time PCR system (Agilent Technologies). We used *Gapdh* expression as an internal control. Primers sequence are available upon request.

### Western Blot Analysis

Proteins were analyzed by SDS/PAGE, and Western blotting was performed according to a standard protocol^37^. The antibodies were anti-CDK1 (Abcam), anti-GAPDH (Sigma Aldrich), and anti-CDK2 (MBL). Proteins were detected using SuperSignal West Dura Extended Duration Substrate (Thermo Scientific). Results are representative of more than four individual experiments.

### Organ culture

Embryonic hind limb organ cultures were performed as previously described^38^ with modifications. In brief, E16.5 limbs were freed of skin and muscles and cultured in BGJ-B medium (Life Technologies) and 0.5% bovine serum albumin in a 24-well cell culture plate for 4 days at 37 °C in a 5% CO_2_ and humidified atmosphere, initially in a medium containing 5% fetal calf serum. After 1 day in culture, the medium was changed to 0.1% FBS, and the right limbs of each embryo were treated with 3 × 10^−7^ M recombinant PTHrP to activate PTHrP signaling, while the left limbs of the same embryo were cultured with PBS as a vehicle control. The experiments were repeated three times. Proliferation was measured using BrdU incorporation. Explant specimens were incubated with BrdU for the final 12 h of culture. Limb explants were then fixed and embedded in paraffin and sectioned at 5 μm for histological and immunohistochemical analysis.

### Statistics

All data are presented as means ± s.e. (*n* ≥ 3). We performed statistical analysis by Student’s *t*-test and *P* < 0.05 was considered statistically significant.

## Additional Information

**How to cite this article**: Saito, M. *et al*. The Indispensable Role of Cyclin-Dependent Kinase 1 in Skeletal Development. *Sci. Rep.* **6**, 20622; doi: 10.1038/srep20622 (2016).

## Supplementary Material

Supplementary Information
