# Supplementary material for: The Indispensable Role of Cyclin-Dependent Kinase 1 in Skeletal Development
Source: Sci Rep. 2016 Feb 10;6:20622. doi: 10.1038/srep20622 (PMC4816159; doi:10.1038/srep20622)
Supplement: Supplementary Information [file srep20622-s1.pdf]

The indispensable role of Cyclin-dependent kinase 1 in skeletal development

Authors: Masanori Saito[1], Mieradili Mulati[1], S. Zakiah A. Talib[2], Philipp Kaldis[2, 3], Shu Takeda[4], Atsushi Okawa[1], and Hiroyuki Inose[1] *

Supplementary Materials

Supplementary text

Fig. S1. The skeletal phenotype of Cdk1 conditional knockout mice

Quantification of tibia length of P0 *Cdk1*$^{f/f}$ and α1(II) *Cdk1*$^{f/f}$ mice. Note the significant decrease in tibial length of α1(II) *Cdk1*$^{f/f}$ mice. *$P < 0.05$, n = 3.

Fig. S2. The effect of *Cdk1* deletion on chondrocyte apoptosis

Chondrocyte apoptosis in *Cdk1*$^{f/f}$ and α1(II) *Cdk1*$^{f/f}$ mice at E17.5 was examined by a TUNEL assay. The number of apoptotic hypertrophic chondrocytes was comparable between groups, suggesting that *Cdk1* has little effect on chondrocyte apoptosis in the growth plate. The TUNEL assay was performed with the ApopTag system (Millipore) according to the manufacturer's instructions.

Fig. S3. The regulative effects of shRNA, siRNA, and pcDNA3.2-Cdk1 on *Cdk1* level.

(a) qPCR analysis of changes in the expression of *Cdk1* in ATDC5 cells after the infection of shRNA against *Cdk1* or negative control. $^*P < 0.05$, n = 4.

(b) qPCR analysis of changes in the expression of *Cdk1* or *Cdk2* in ATDC5 cells after the treatment with siRNA against *Cdk1* or *Cdk2*. $^*P < 0.05$, n = 4.

(c) qPCR analysis of changes in the expression of *Cdk1* in ATDC5 cells after the transfection with pcDNA3.2-Cdk1 or control. $^*P < 0.05$, n = 4.

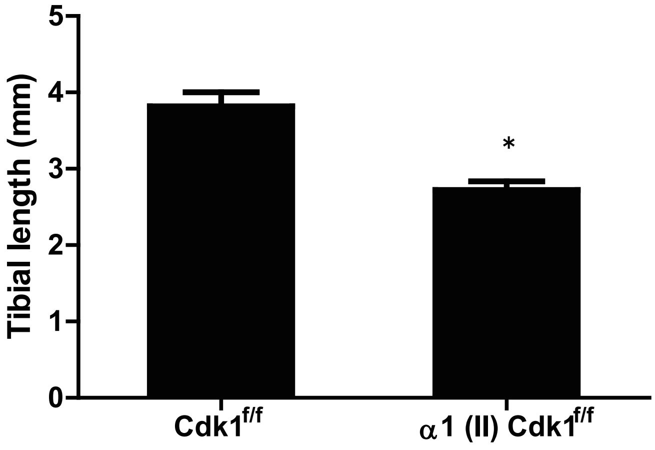

Tibial length (mm)
5
4
3
2
1
0
*
Cdk1f/f
α1 (II) Cdk1f/f

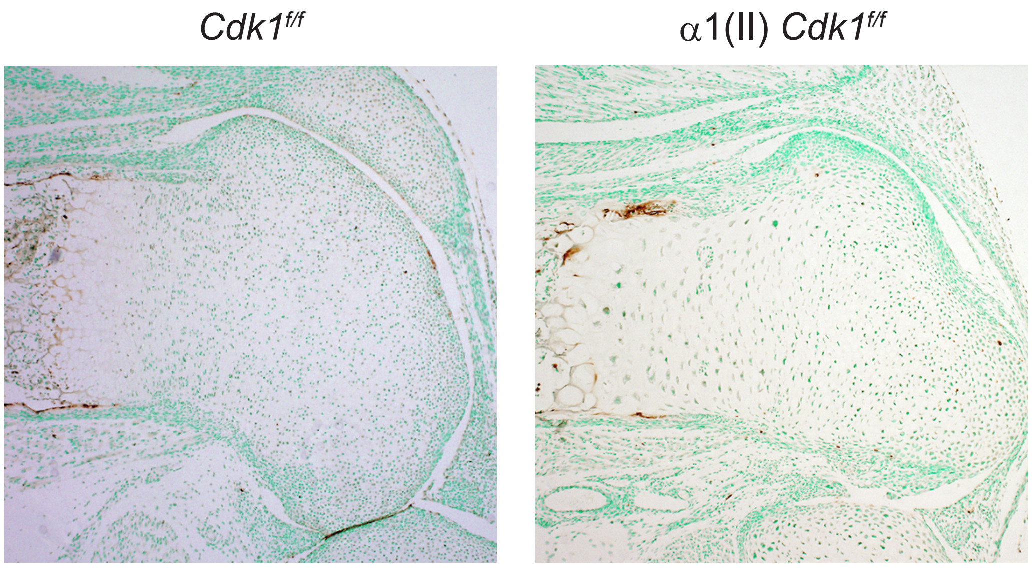
Cdk1$^{f/f}$
α1(II) *Cdk1*$^{f/f}$

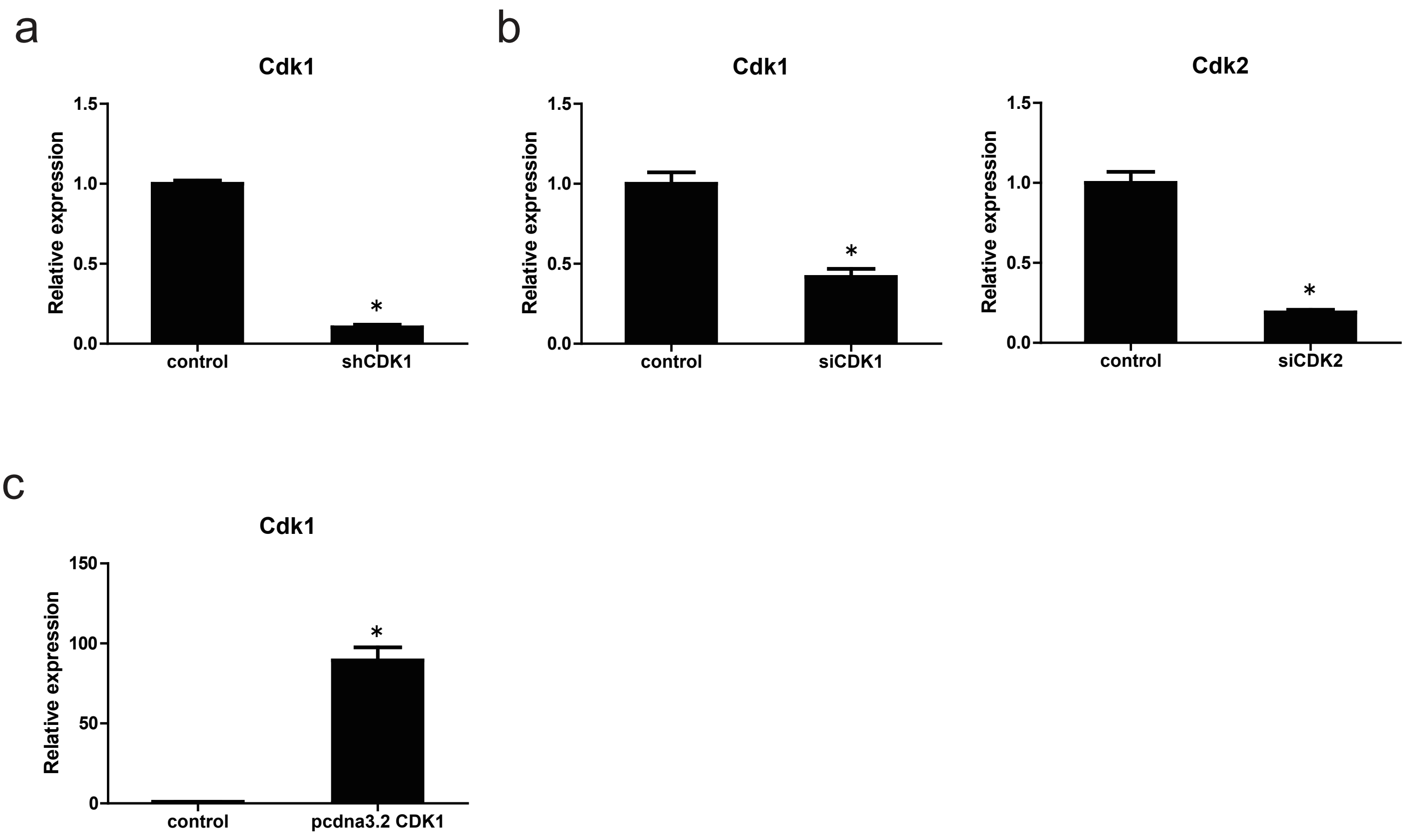

a
Cdk1
Relative expression
1.5
1.0
0.5
0.0
*
control
shCDK1
b
Cdk1
Relative expression
1.5
1.0
0.5
0.0
*
control
siCDK1
Cdk2
Relative expression
1.5
1.0
0.5
0.0
*
control
siCDK2
c
Cdk1
Relative expression
150
100
50
0
*
control
pcdna3.2 CDK1
